# No Fault Compensation for Accidental Injury

**Published:** 1986-08

**Authors:** Gordon M. Stirrat

**Affiliations:** Professor of Obstetrics and Gynaecology, University of Bristol


### No Fault Compensation for Accidental Injury

**Gordon M. Stirrat, M.A., M.D., F.R.C.O.G.**
Professor of Obstetrics and Gynaecology, University of Bristol

The numbers of claims for medical mishap has increased dramatically in recent years and the total amount paid annually to successful claimants has increased at least tenfold in the last fifteen years. No-one will dispute that high standards of care should be encouraged and basic minima enforced, but the present situation ultimately benefits only the lawyers.

It has been projected that, if the present trend continues, the annual subscription to our medical defence societies (currently £336) will rise to £1000 within the next three years.

Health authorities, unable to insure against medical mishap must pay lost suits from current revenue. It is not uncommon for awards to exceed £500,000 with costs. How many such are needed to knock District and Regional financial plans irrevocably off an already constrained course?

The problem is, of course, perceived differently by the doctor and the hapless victim of medical mishap or malpractice, but there is agreement that the present situation is unacceptable. A meeting was, therefore, held recently in London by the Medical Education and Information Unit (MEIU) of the Spastics Society at which all aspects of the problem were discussed. A list of participants is appended. What follows is a synthesis of that discussion.

### The Nature of the Problem

The basic problem lies in the fact that the British legal system is adversarial. In cases of medical mishap the doctor must have been shown to be negligent for the victims to obtain compensation. It is therefore inevitable that such terms as 'fault' and 'blame' are used, and, as a result the medical profession's stance becomes defensive.

At the root of the problem lies the question of communication with its several components and confounding factors. For example:

1. The expectation of patients is extremely high—often unrealistically so.
2. The sense of a balance between risk and benefit is often not communicated by the doctor and/or adequately perceived by the patient.
3. The patient's sense of a doctor's infallibility may be a required part of the healing process.
4. The person seeking medical care is, by definition, in a vulnerable state. Once 'something has gone wrong' the anger and grief of the patient or his family can produce a *need* to find fault.
5. The anatomy of medical mishap is complex. Hushed voices at the end of the bed or lack of clear and concise information, which may be due to uncertainty among doctors or nurses, are perceived as conspiracy by the aggrieved.
6. In cases which involve handicapped children (the area of primary interest to the Spastics Society), arguments on causation are often based on flimsy and simplistic evidence. Doctors are unwilling to admit (and patients refuse to accept) that they 'do not know'.

### The Extent of the Problem

The problem is much bigger than is generally acknowledged. Statistics are kept by the medical defence societies but are not divulged. It is known that in 1981 700 legally aided writs were served for medical negligence and it is likely that that number has increased at least four-fold since then. The number of cases being brought in relation to perinatal brain damage has increased dramatically in recent years.

In an estimated 70% to 80% of cases the patient or his family are not seeking damages but satisfaction and information. They want to see that 'the same thing does not happen again'. In approximately 20% to 25% of cases the negligence is clear. Only a tiny minority of all claims (ca 1%) are fought out in court.

### The Patient's Viewpoint

Those injured by medical mishap face many problems in pursuing any action, however justified. Among them are:

1. The cost involved—only the incredibly wealthy or those assisted by legal aid can afford it.
2. Finding an experienced lawyer—the patient is likely to approach the lawyer who, for example, helped with house purchase. Such 'general practitioner' lawyers are inexperienced in medical negligence cases and are, therefore, at a disadvantage compared to the experts used by the medical defence societies.
3. Finding a medical expert to prepare a report—some members of the medical profession feel that it is 'not quite the done thing' to act on behalf of the plaintiff. The call on the time of those willing to do so in pre-trial preparations and particularly in court is so great as to be a severe disincentive.
4. The length of time before settlement—the case can drag on for many years. This is sometimes due to stalling by health authorities (it is amazing how often the plaintiff's case-notes are inexplicably lost for several years) or others, but can be due to the time taken before the full nature of residual damage can be assessed.

### The effect of continuing as we are

Although some believe that 'patients are turning against the profession', this is not generally accepted. The nature of the 'social contract' is, however, changing. (For discussion of this, see Editorial, J. Med. Ethics. 1985, **11**, 59–60). As a result the practice of 'defensive medicine' is increasing. This has 'knock-on costs' which can be serious; for example, the increasing recourse to caesarean section, in the unproven belief that it is safer for the child, produces greater risk of morbidity and mortality to the mother without necessarily reducing the incidence of cerebral palsy in the offspring.

Although we are told that 'our view should not be clouded by the American scene' this is the spectre which is in the minds of many in the medical profession. Rumours of differential subscriptions to medical defence societies for doctors in 'high risk' specialties, e.g. obstetrics, orthopaedics, neonatology and neuro-surgery, has fuelled these fears. In the interests of all concerned, every effort should be made to prevent the growth in adversarial litigation. Any alternative needs to provide information on causation and a decision on compensation. The initial effort should be concentrated on changing attitudes within the medical profession.

**Alternative approaches**

The value of the Confidential Inquiry into Maternal Mortality (HMSO), which has produced triennial reports since 1958, is generally acknowledged. A similar system should be instituted for perinatal mortality and, perhaps, morbidity.

The problem of injury due to medical mishap is much smaller and less complex than those of industrial or criminal injury, yet both of these are being dealt with satisfactorily through relevant compensation boards backed up by appeals and judicial reviews. It is important to move away from thoughts of 'compensation for' to 'insurance in case of' an injury due to medical mishap. If this could be achieved, the process of informing would be separated from incrimination.

Japan, Germany and Israel have schemes to 'compensate' for medical mishap but the two which were discussed in greater detail and are summarised here relate to Sweden and New Zealand.

**Swedish system**

Each county council contributes to a common pool on a per capita basis. A panel of six members assesses each case and indemnity is paid according to agreed scales. The concept of negligence does not exist any longer and investigation of causation is not encouraged.

If such a system were introduced into the UK, the Swedish experience suggests it would assess about 13,000 cases per annum and cost approximately £20 milion. The main criticism of this system is that it concentrates on monetary compensation without addressing the question of standards of practice. Even a straight insurance system would do the same. Negligence must not be swept under the carpet. Confidential enquiry into causation needs to coexist but be separate from assessment of 'compensation'.

**New Zealand Scheme**

The details of this scheme are complex. Its aim is to compensate personal injury by accident. Many of the problems hinge around the definition of medical mishap. Although one object of the scheme was to replace litigation for medical negligence with non-contentious processes of assessment, it is suggested that this has not happened. The arguments have moved into the realm of appeal and legal review, thus providing 'a field-day for lawyers'—exactly the opposite of what was proposed.

**The Way Forward**

The following should be among the principles behind any scheme proposed for the UK.

1. Contentious words such as 'fault' and 'compensation' should be set to one side.
2. The process of informing should be separate from incrimination.
3. Any proposed scheme must initially confine itself to cases of medical mishap or other injury arising from treatment or investigation.
4. This could be financed through an insurance scheme (either private or state funded) in which individual patients participate against remote risks of high impact. The medical profession's insurance scheme would continue.

Energetic attempts must be made to move discussion of this important topic into the public domain so that justice can be, and can be seen to be, achieved for all parties involved.

**Participants**

| | |
|---|---|
| Dr Martin Bax | Spastics International Medical Publications, London |
| Mrs Diana Brahams | Barrister, Lincoln's Inn |
| Miss Diana Elbourne | National Perinatal Epidemiology Unit, Oxford |
| Dr Aidan Macfarlane | Department of Community Health, Oxford |
| Dr Brian Neville | M.E.I.U., Guy's Hospital |
| Mrs Diana Patterson | M.E.I.U., Guy's Hospital |
| Mrs Margaret Puxon | Q.C., Temple |
| Dr Ian Quest | Medical Protection Society |
| Dr Martin Robards | Consultant Paediatrician, Tunbridge Wells |
| Mr Arnold Simanowitz | Director, Action for the Victims of Medical Accidents |
| Professor Gordon Stirrat | Department of Obstetrics and Gynaecology, University of Bristol |
| Professor David Taylor | Department of Child and Adolescent Psychiatry, Manchester |

